# Comparative chemosensory cognition

**DOI:** 10.3389/fnbeh.2014.00190

**Published:** 2014-05-23

**Authors:** Alan Gelperin

**Affiliations:** Department of Molecular Biology, Princeton Neuroscience Institute, Princeton University, PrincetonNJ, USA

**Keywords:** comparative cognition, olfactory learning, invertebrate consciousness, higher order conditioning, genetic model systems

Don Griffin, one of the foremost students of comparative cognition, offered the following definition of cognition: “The term cognition is ordinarily taken to mean information processing in human and nonhuman central nervous systems that often leads to choices and decisions” (Griffin and Speck, 2004). Clearly we can study cognition by analyzing animal information processing systems and the manner in which behavioral choices and decision making are altered by experience. The use of olfactory information for guiding a wide range of basic biological decisions is ubiquitous in animals, including humans (Gelperin, 2010). Chemosensory processing, and particularly olfactory information processing, is a particularly attractive modality within which to seek comparative insights into cognitive processes underlying learning and memory.

The advent of modern molecular and genetic tools for selectively modifying and perturbing functionally identified groups of neurons, particularly optogenetic methods, has led to a focus on chemosensory processing in a limited number of species for which genetic tools are well developed, including *C. elegans* (Glater et al., 2014) and *D. melanogaster* (Wilson, 2013), among others. Looking more broadly for instances of chemosensory learning reveals a remarkable diversity of cognitive plasticity from ants to zebrafish. The renewed interest in comparative cognition (Shettleworth, 2012) plus glimmerings of recognition that a voluminous and highly folded cerebral cortex is not required for implementation of higher order logic operations (Watanabe et al., 2008) is producing renewed interest in exploration of chemosensory processing and learning in an increasingly diverse set of species.

Another impetus for comparative studies is the quest to identify the minimum essential neural circuit that can implement the synaptic operations required for higher-order learning and decision making involved in choosing among alternative neural outputs based on previously learned weighting factors, as in reinforcement learning (Wilson et al., 2014). Modeling studies tightly constrained by demonstrated or plausible neuronal and synaptic properties are essential to this enterprise, having been used to show for example that five neurons with suitable dynamics and plausible synaptic plasticity functions can demonstrate the Kamin blocking effect, previously shown behaviorally in *Limax* (Goel and Gelperin, 2006). In the face of overwhelming evidence that neuronal properties and synaptic communication are essentially similar from ants to zebrafish (Llinas, 2008), the hope is that gaining new insights into the minimum essential circuitry for higher order information processing functions may be more straightforward in compact nervous systems comprised of fewer computing elements compared to the mammalian central nervous system. The use of transgenic *Drosophila* mutants as models of human disease (Chen and Crowther, 2012; van Alphen and van Swinderen, 2013) is also fueling this enterprise.

A remarkable example of olfactory learning in a compact brain is the demonstration of food imprinting and prenatal chemosensory conditioning in the predatory mite *Neoseiulus* (Peralta Quesada and Schausberger, 2012), whose entire brain of 10,325 cells occupies a single synganglion, containing a prominent olfactory lobe, measuring only 100 by 65 microns (van Wijk et al., 2006). A more familiar example of a compact brain with excellent olfactory abilities is that of the no-see-um, e.g., *Culicoides sonorensis*, best described as a flying nose with gonads and biting mouthparts measuring only a few hundred microns in overall body size. The success of these tiny cognitive engines is shown by the fact that the family containing the no-see-ums, the Ceratopogonidae, contains over 4000 species worldwide. No-see-um olfactory learning is likely as members of a species of larger size, also with obligate blood-sucking by females, are know to modulate their olfactory responses due to associative learning (Sanford et al., 2013).

Consider the noble nematode, *C. elegans*, possessed of 302 neurons, only a subset of which is needed to implement associative chemosensory conditioning modulated by dopamine (Mersha et al., 2013). Dopamine is a familiar neuromodulator in the mammalian CNS implicated in the processing of signals and synapses mediating reward and expectation (Kobayashi and Schultz, 2014). *C. elegans* possesses multiple learning types modifying its information processing in non-chemosensory modalities (Ardiel and Rankin, 2010). This suggests that a search for higher order conditioning such as second order conditioning and blocking using chemosensory stimuli might be fruitful.

A detailed prescription for comparing cognitive abilities across wide phyletic boundaries was provided by Bullock (1993), who emphasized the need for quantitative measurement of higher order cognitive operations by invertebrate brains. He also highlighted the need to frame experimental questions assessing complex information processing with special regard to the neuroethological context within which experimental questions are most effectively and insightfully asked of the animal subject. The use of olfactory stimuli to ask questions about cognitive aspects of higher order learning has been particularly fruitful for terrestrial gastropods in general (Gelperin, 2013) and *Limax maximus* in particular (Watanabe et al., 2008). *Limax* has been shown to be capable of a variety of higher order learned logic operations on olfactory stimuli, most recently involving the ability to learn the association between olfactory stimuli and the reward of access to water after the animal was subjected to rapid and severe dehydration, a normal stressor for terrestrial slugs. Olfaction is the dominant sensory modality for distance perception in terrestrial mollusks and a brain region with unique neuronal architectures and dynamics, the procerebral lobe, is devoted to learning about odors (Matsuo et al., 2011). Like the mushroom bodies of insects and the vertical lobes of octopi, identification of the sensory inputs to these distinctive central information processing centers in invertebrates can help guide the choice of sensory modalities within which to look for complex cognitive logic operations.

The cognitive abilities of honeybees in the chemosensory domain include not only higher order chemosensory learning, but also the construction and use of cognitive maps incorporating multiple domains of sensory input during their construction, particularly visual and chemosensory inputs (Gould, 1990). In 1973 Karl von Frisch shared the Nobel Prize for his pioneering work on the multimodal communication methods used by bees in communicating and receiving information on potential food sources in the environment surrounding the hive. Several generations of von Frisch's scientific descendants have continued his neuroethological tradition in selecting behavioral questions arising from the use of natural odor, taste, color, and shape information contained in the floral fountains exploited by bees. Processing of these proximate cues is interlaced with the processing of visual and magnetic information for navigation from hive to food source. Thus, chemosensory cues are only one aspect of a multimodal processing system that is capable of concept learning, a form of higher order learning that relies on the relationships between objects (e.g., same/different, left/right, above/below) rather than the specific properties of individual stimuli. These studies used the training protocols of delayed matching to sample and delayed non-matching to sample (Reinhard et al., 2004), protocols that are widely used for probing cognitive aspects of stimulus representation in primates. A recent summary of the cognitive architecture of the honeybee brain, which contains 950,000 neurons packaged in a volume of 1 mm^3^, identifies more than 17 discrete categories of computations as demonstrated abilities of the honeybee brain, including a requirement for neuronal circuitry tasked with assigning value to stimulus configurations, a value assignment that changes with experience (Menzel, 2001). Another mammalian cognitive parallel is the demonstration that honeybees consolidate a novel navigation memory during sleep (Menzel and Giurfa, 2001).

Chemosensory cognition in *Drosophila* has only recently come under experimental examination, although the study of olfactory learning is well developed (Young et al., 2011; Beyaert et al., 2012), augmented by experimentally useful modeling work (Wessnitzer et al., 2012). The seminal initial work on *Drosophila* learning was done by molecular biologists (Quinn et al., 1974) so work focused initially on development of high throughput screens for memory mutants rather than identification of natural stimulus configurations and contingencies that would provide natural experimental approaches to asking cognitive questions (Tomchik and Davis, 2013). Nonetheless in more recent work behavioral and neurobiological aspects of sleep, dopaminergic arousal, aggression, selective attention and courtship in *Drosophila* have been identified. Both aggression and courtship in *Drosophila* have critical chemosensory components involving both olfactory and gustatory receptors that allow male flies to distinguish between potential mates and competing conspecific males (Hollis and Kawecki, 2014). The maintenance of a range of cognitive tasks in male *Drosophila*, including but not limited to olfactory learning ability, was significantly reduced after 100 generations of enforced monogamy (Anderson and Adolphs, 2014).

Another example of a genetic model system that has engendered work on olfactory cognition is the zebrafish, *Danio* (Friedrich, 2013). Odors are known to be critical cues for guiding a number of behaviors in fish, including homing, reproduction, ingestion and social and avoidance behaviors. Aqueous odors can also participate in eliciting food-aversion conditioning, accompanied by induction of the immediate early response gene Egr-1 in gustatory areas of the zebrafish brain (Boyer et al., 2013). High throughput methods have been developed for assessment of the effects of genetic and pharmacological manipulations on visual responses of larval zebrafish behavior. Extension of these methods to olfactory conditioning will allow assessment of olfactory learning in a quantitative and unbiased fashion and facilitate the search for higher order learning about odors.

The power of genetic tools has promoted a focus on a limited number of animal species, known as genetic model systems, that enable use of the so-called genetic toolbox. This trend is augmented by multiple demonstrations that human disease genes and their downstream effects can be usefully studied in some of these genetic model systems, particularly *Drosophila*. Work on comparative cognition among vertebrate species provides more and more examples of cognitive skills among non-hominids, particularly but not exclusively elephants, birds and cetaceans. An interesting generalization from this effort is to look for further examples of higher order learning and other cognitive skills among invertebrates. For example, the debate on whether *Octopus* is conscious has already begun (Mather, 2008), fueled by descriptions of unique personality types among captive specimens (Mather and Kuba, 2013).

The unique relationship between neural circuits processing olfactory memory and circuits controlling emotions may provide yet another unique vantage point for a comparative approach to chemosensory cognition (Anderson and Adolphs, 2014). The proposal stresses the common features and evolutionary advantages of modes of behavior that are commonly identified as outward manifestations of emotions. An example is provided by negative or positive odor conditioning in male *Drosophila*, where flies show a conditioned positive place preference to an odor previously paired with mating with a virgin female (Shohat-Ophir et al., 2012). Mating responses to virgin females require neuropeptide Y, also involved in reward learning to ethanol. The concatenation of these findings suggests that these learned responses involve a rewarding internal state, a potential substrate for positive emotions. The idea that certain brain states can be rewarding, as indexed by their ability to promote the increased probability of discrete behaviors with which they are associated, is also supported by the finding that direct electrical stimulation of discrete brain areas in the cerebral ganglion of the terrestrial snail *Helix* can lead to significant increases in the probability of occurrence of the behaviors yoked to the application of brain stimulation (Balaban and Chase, 1989; Balaban and Maksimova, 1993). The use of implanted electrodes in minimally restrained animals (Cooke and Gelperin, 2001) increases the range of possible analyses of the rewarding effects of direct brain stimulation.

If the concept of consciousness loses its uniquely mammalian brand, the study of olfactory information processing may be the most general and fruitful approach to the study of comparative cognition, including consciousness, in the 96% of animal species in the Invertebrata. Some invertebrates could have a brain state representing a precursor of consciousness, as recently suggested for *Drosophila* (van Swinderen, 2005). Thus, understanding chemosensory cognition could help unravel some of the mechanisms underlying an evolutionary precursor to hominid consciousness.

This effort was presaged by Vince Dethier in his paper on Microscopic Brains, which ends with the following: “Perhaps these insects are little machines in a deep sleep, but looking at their rigidly armored bodies, their staring eyes, and their mute performances, one cannot help at times wondering if there is anyone inside” (Dethier, 1964).

## Conflict of interest statement

The author declares that the research was conducted in the absence of any commercial or financial relationships that could be construed as a potential conflict of interest.
